# Pandemic Leadership: Sex Differences and Their Evolutionary–Developmental Origins

**DOI:** 10.3389/fpsyg.2021.633862

**Published:** 2021-03-15

**Authors:** Severi Luoto, Marco Antonio Correa Varella

**Affiliations:** ^1^English, Drama and Writing Studies, University of Auckland, Auckland, New Zealand; ^2^School of Psychology, University of Auckland, Auckland, New Zealand; ^3^Department of Experimental Psychology, Institute of Psychology, University of São Paulo, São Paulo, Brazil

**Keywords:** COVID-19, sex differences, cognition, leadership, pandemic, population health, evolution, sexually dimorphic leadership specialization

## Abstract

The COVID-19 pandemic has caused a global societal, economic, and social upheaval unseen in living memory. There have been substantial cross-national differences in the kinds of policies implemented by political decision-makers to prevent the spread of the virus, to test the population, and to manage infected patients. Among other factors, these policies vary with politicians’ sex: early findings indicate that, on average, female leaders seem more focused on minimizing direct human suffering caused by the SARS-CoV-2 virus, while male leaders implement riskier short-term decisions, possibly aiming to minimize economic disruptions. These sex differences are consistent with broader findings in psychology, reflecting women’s stronger empathy, higher pathogen disgust, health concern, care-taking orientation, and dislike for the suffering of other people—as well as men’s higher risk-taking, Machiavellianism, psychopathy, narcissism, and focus on financial indicators of success and status. This review article contextualizes sex differences in pandemic leadership in an evolutionary framework. Evolution by natural selection is the only known process in nature that organizes organisms into higher degrees of functional order, or counteracts the unavoidable disorder that would otherwise ensue, and is therefore essential for explaining the origins of human sex differences. Differential sexual selection and parental investment between males and females, together with the sexual differentiation of the mammalian brain, drive sex differences in cognition and behavioral dispositions, underlying men’s and women’s leadership styles and decision-making during a global pandemic. According to the *sexually dimorphic leadership specialization hypothesis*, general psychobehavioral sex differences have been exapted during human evolution to create sexually dimorphic leadership styles. They may be facultatively co-opted by societies and/or followers when facing different kinds of ecological and/or sociopolitical threats, such as disease outbreaks or intergroup aggression. Early evidence indicates that against the invisible viral foe that can bring nations to their knees, the strategic circumspection of empathic feminine health “worriers” may bring more effective and humanitarian outcomes than the devil-may-care incaution of masculine risk-taking “warriors”.

## Introduction

The novel coronavirus and the disease that it causes (i.e., COVID-19) created a social and economic upheaval unseen in the past half a century or more. The political and social responses to the COVID-19 pandemic, as well as the SARS-CoV-2 virus itself, have both had major effects on economic activity, public policy, civic engagement, and population health almost all over the world (Bedford et al., 2020; Weible et al., 2020). Being under direct human control, such policy responses (versus inaction) have the potential to diminish the impact of the virus or to amplify its disastrous effects.

**FIGURE 1:**
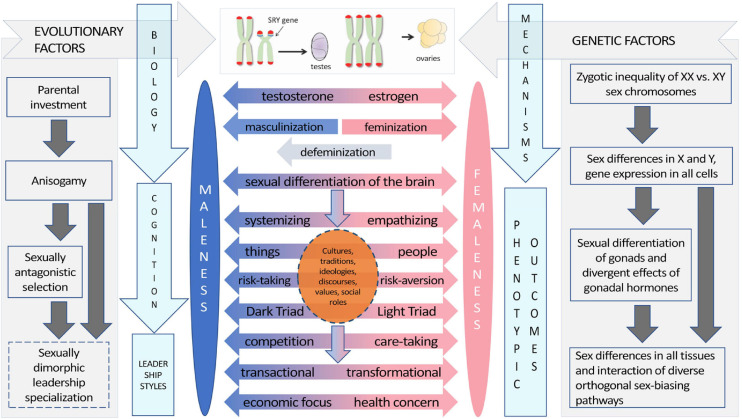
The evolutionary–developmental origins and proximate mechanisms underlying psychobehavioral sex differences, including those in leadership. Sexually dimorphic leadership specialization is included as a new hypothesis, not as an established fact. Figure adapted from Luoto et al. (2019a), Arnold (2020), McCarthy (2020), and Malmi (2021).

We review the evidence on cross-national differences between male and female leadership during the pandemic and discuss the possible evolutionary–developmental and psychobehavioral mechanisms underlying such differences (Figure 1). Based on a review of relevant research in evolutionary science, psychology, behavioral science, anthropology, political science, economics, behavioral genetics, and developmental, cognitive, and behavioral neuroscience, we also present the *sexually dimorphic leadership specialization hypothesis* as one of the possible explanations for these cross-national patterns.

## Public Policy Responses to the COVID-19 Pandemic

An increased consensus has emerged on how to effectively manage the COVID-19 pandemic and the transmission of the SARS-CoV-2 virus (Habersaat et al., 2020; Kaplan et al., 2020; Priesemann et al., 2021). Countries have implemented a range of measures to curb the spread of the virus (Bedford et al., 2020); while some countries have implemented strict measures that have shut public life and most commercial activity almost completely, others have kept significant parts of society open even though faced with similar health threats imposed by SARS-CoV-2. Research into the factors that predict cross-national differences in pandemic responses and subsequent outcomes has been conducted during the pandemic’s global spread (Coelho et al., 2020; Puterman et al., 2020; Salvador et al., 2020), and among other factors (cf. Burkle, 2020; Windsor et al., 2020; Krams et al., 2021), political leaders’ sex hypothetically contributes to cross-national variation in pandemic outcomes.

## Sex Differences in Pandemic Leadership and Cross-National COVID-19 Outcomes

To provide a prominent example, Brazil’s President Jair Bolsonaro has mostly downplayed the COVID-19 health threat and has implemented less severe societal measures than many other political leaders in the first months of the pandemic (Ponce, 2020). When asked about the rapidly rising cases of COVID-19 victims in Brazil in May 2020, President Bolsonaro responded with a callous “So what? What do you want me to do?”, whilst continuing to flout and discourage physical distancing and lockdown policies (Prado, 2020). When infected with SARS-CoV-2, he broke quarantine regulations to ride a motorcycle and interacted mask-less with people. With 746 COVID-related deaths per million inhabitants by October 30th 2020, Brazil was ranked the country with the 6th most COVID-related deaths (for details, see Supplementary Materials).

Other political leaders have taken the opposite approach. New Zealand implemented draconian lockdown measures at a stage when there were only 102 confirmed COVID-19 cases and no reported deaths on March 23rd 2020. The Prime Minister, Jacinda Ardern, emphasized the importance of early, preventative action in her address to the citizens of New Zealand on the eve of societal lockdown: “act now, or risk the virus taking hold, as it has elsewhere […] the situation here is moving at pace, and so must we […] together, we must stop [the virus from spreading and killing tens of thousands of New Zealanders]. Now is the time to act” (Ardern, 2020). Her approach was so successful that her popularity skyrocketed, leading to a landslide victory for her party in the New Zealand parliamentary election in October 2020. With five COVID-related deaths per million inhabitants by October 30th 2020, New Zealand had one of the lowest mortality rates globally (Supplementary Materials).

Garikipati and Kambhampati (2020) examined the association between political leaders’ sex and variation in pandemic responses and outcomes across 194 countries (19 of which were coded as female-led). Female leaders, on average, reacted more quickly and decisively to the COVID-19 pandemic than their male counterparts, implementing measures that resulted in lower mortality rates (Garikipati and Kambhampati, 2020). These results remained robust when controlling for country-level annual health expenditure, openness to tourists, Gender Inequality Index (a measure of women’s versus men’s participation in politics and the labor force), per capita gross domestic product (GDP), population size, urbanization, and population over 65 years of age. This preliminary analysis was based on total deaths and total cases due to COVID up to May 19th 2020, and therefore covered only the first months of the pandemic. However, a cross-national study including 15 female-led countries found no country-level differences based on leaders’ sex in time to implementation for any of the most common COVID-19 containment policies: stay-at-home orders, school closings, public information campaigns (Aldrich and Lotito, 2020), indicating that female leaders were (statistically) no quicker than male leaders to implement such measures. Nevertheless, 63% of women-led countries, as opposed to only about half of all countries, launched coordinated information campaigns before their first confirmed case of COVID-19, and average time to implementation was one week shorter in women-led countries than in male-led ones (Aldrich and Lotito, 2020).

**FIGURE 2:**
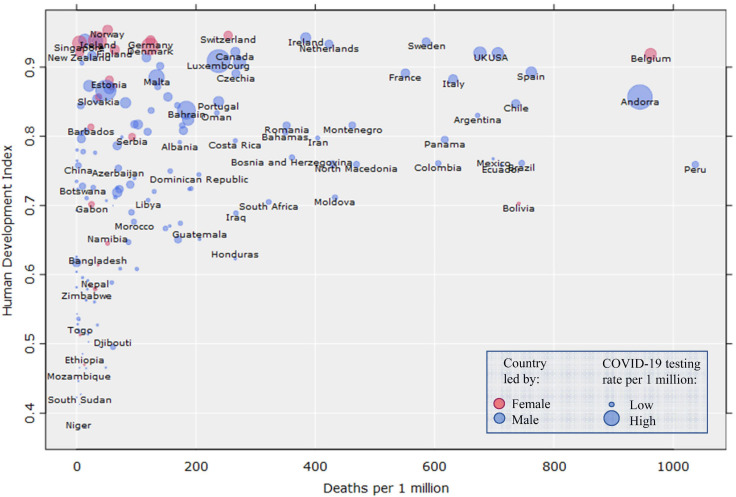
COVID-19 deaths per 1 million in relation to the Human Development Index (*N* = 168, *r*_*s*_ = 0.51). Data points are colored according to the sex/gender of the country leader, and scaled to COVID-19 tests per 1 million. For the full data, see Supplementary Materials.

**FIGURE 3:**
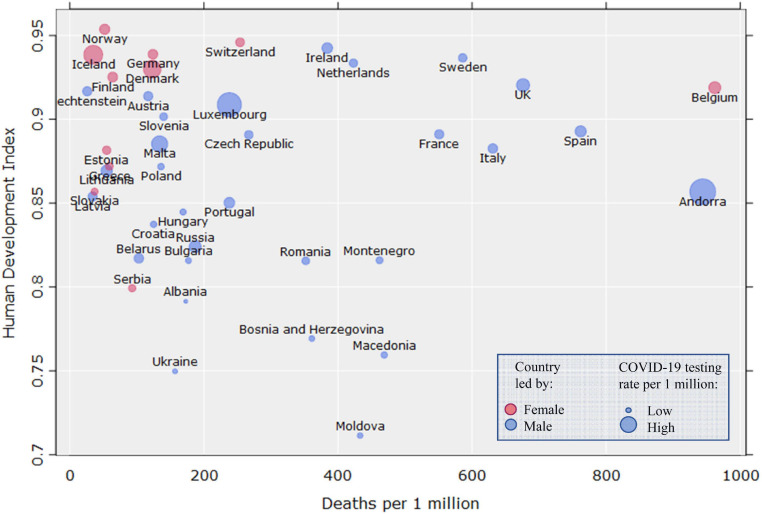
COVID-19 deaths per 1 million in relation to the Human Development Index in European countries (*n* = 41, *r*_*s*_ = –0.07). Data points are colored according to the sex/gender of the country leader, and scaled to COVID-19 tests per 1 million.

Another study of 159 countries found that female-led countries had lower median case-fatality rates relative to male-led countries through June 3rd 2020; however, because of the small sample of female-led countries (*n* = 18), the difference did not reach conventional levels of statistical significance (Purkayastha et al., 2020). The results are nevertheless suggestive^1^. These patterns in male-led and female-led countries are visualized in Figure 2, which shows global COVID-19 deaths per 1 million inhabitants as a factor of the Human Development Index (HDI), using more recent data than Purkayastha et al. (2020) and Garikipati and Kambhampati (2020)^2^. The data points are colored to reflect the sex of each country’s leader, and scaled according to COVID-19 testing rates per 1 million inhabitants. For higher granularity, Figure 3 shows the same outcomes only in Europe^3^.

The relationship between leader’s sex and a population’s COVID-19 outcomes has also been studied at the level of states in the United States. As of May 5th 2020, states (*N* = 55, comprising 50 states, the District of Columbia, and four American Territories) with female governors had fewer COVID-19 deaths than states with male governors (Sergent and Stajkovic, 2020). States with women governors who issued early stay-at-home orders also had fewer deaths compared to states with men governors who issued similar orders (Sergent and Stajkovic, 2020). The study controlled for governor’s political affiliation (Sergent and Stajkovic, 2020), but not for other biodemographic variables such as state-level rates of obesity, smoking, or age structure, which could all have influenced differences in COVID-19 outcomes between states (Jordan et al., 2020; Krams et al., 2020). The results are nevertheless in line with the cross-national data. Furthermore, a psycholinguistic analysis of 251 briefings from 38 different state governors comprising 1.2 million words indicated that female governors, relative to male governors, showed more empathy via greater awareness of the feelings of others (Sergent and Stajkovic, 2020). Female governors also spoke more about work and money, perhaps to reassure followers that there is a brighter future ahead. South Dakota Governor Kristi Noem, for instance, noted in her address on April 6th 2020 that “resources are available to you, whether it be economic or mental health and labor unemployment” (Sergent and Stajkovic, 2020)^4^.

Generally, some of the leaders who have shown the strictest, most humanitarian responses to the pandemic are females (e.g., Jacinda Ardern in New Zealand, Katrín Jakobsdóttir in Iceland, Sanna Marin in Finland), while the most indifferent or even reckless responses have been made by male leaders (e.g., Jair Bolsonaro in Brazil and Stefan Löfven and the state epidemiologist Nils Anders Tegnell in Sweden) (Figures 2, 3). The 2020 Ig Nobel prize in “Medical Education” was awarded to a group of male political leaders “for using the Covid-19 viral pandemic to teach the world that politicians can have a more immediate effect on life and death than scientists and doctors can” (Tanne, 2020)^5^. The world leaders edition of the *BMJ* “COVID-19 yearbook” also confirms this same pattern (Looi, 2020). Overall, leadership style, communication, and policy-making during pandemics are important for population-level outcomes because trust in authorities has a positive effect on the adoption of many protective behaviors (Gong et al., 2020; see also Haslam et al., 2021).

## Psychobehavioral Sex Differences

Psychological sex differences, such as men’s higher risk-taking, systemizing, and things orientation—and women’s higher fearfulness, empathizing, and people orientation—have been reported in a variety of domains (Geary, 2010; Christov-Moore et al., 2014; Varella et al., 2016; Greenberg et al., 2018; Archer, 2019; Luoto, 2020), and may be instrumental in decision-making in a high-pressure leadership context (Sweet-Cushman, 2016). The multivariate space of personality differences between men and women has been measured as *D* = 2.71 (in a US sample), corresponding to an overlap of only 10% between male and female personality profiles, assuming statistical normality (Del Giudice et al., 2012). In some cases, male and female political leaders’ responses to the COVID-19 pandemic may reflect a similar kind of difference. We should also note, however, that such generalizations represent average sex differences and that individual variation within each sex tends to be larger than differences between the sexes (Archer, 2019; Del Giudice, 2019; Luoto et al., 2019a). It is also possible that executive positions have a homogenizing effect on personality whereby psychologically more male-typical women pursue and are chosen for leadership positions (Wille et al., 2018). We return to these issues at the end of this article after reviewing research on psychobehavioral sex differences in this section, and their evolutionary–developmental origins in the next section.

### Personality

Systemizing–empathizing is a sexually dimorphic cognitive dimension which is highly relevant to leadership and decision-making. *Systemizing* refers to the tendency to build a rule-based system, to see patterns in systems, and/or to understand how such rule-based systems work. *Empathizing* refers to the ability to recognize another person’s mental state (“cognitive empathy”) and the tendency to respond to it with an appropriate emotion (Greenberg et al., 2018; Archer, 2019). Men tend to score higher on systemizing (Cohen’s *d*s are generally medium to very large, ranging between 0.31 and 1.21), while women, on average, have higher scores on empathizing (Cohen’s *d*s generally ranging between –0.39 and –0.87) (Greenberg et al., 2018; Archer, 2019; see also Löffler and Greitemeyer, 2021).

Sex differences in people and things orientation are found across various psychobehavioral domains. Findings consistently show that women, on average, perceive and orient toward people with greater psychological interest, whereas men, on average, are psychobehaviorally more oriented toward objects than women are (Su et al., 2009; Archer, 2019; Luoto, 2020). The degree to which men and women differ in the psychological salience of people vs. objects (*d* = –0.93 in a meta-analysis), and how it affects men’s and women’s behavior and decision-making (Archer, 2019; Luoto, 2020), is relevant in a pandemic leadership context. These psychological sex differences may make cautious, humanitarian responses more natural to female leaders, while male leaders may be more concerned with retaining the integrity of the socioeconomic system.

There are several other personality differences between men and women which may make female leaders’ responses to the pandemic more humanitarian. On average, men have lower fear in real-world situations (*d* = –1.16), lower social interests (*d* = –0.68), social leadership (*d* = –0.18), peer attachment (*d* = –0.51), guilt (*d* = –0.27), and emotional intelligence (*d* = –0.47) than women (Archer, 2019). Across cultures, women have higher average levels of neuroticism (the tendency to experience negative emotions) than men, with overall effect sizes averaging *d* = –0.40 (Schmitt et al., 2008; Kajonius and Johnson, 2018; Archer, 2019). Women also tend to exhibit higher anxiety (*d* = –0.59), agreeableness (*d* = –0.29), and conscientiousness (*d*s from –0.12 to –0.21)^6^ than men (Schmitt et al., 2008; Archer, 2019; Allen and Robson, 2020).

More neurotic individuals tend to be hypervigilant, experience anticipatory anxiety, and threat sensitivity (Barlow et al., 2014). More agreeable individuals tend to exhibit higher altruism, tender-mindedness, and health consciousness. Individuals with high conscientiousness exhibit self-discipline, are aware of their responsibilities toward society, and show more health consciousness (Kaynak and Ekşi, 2014). Neuroticism, agreeableness, and conscientiousness are among the personality traits related to compliance with the shelter-in-place measures during the COVID-19 pandemics (Götz et al., 2020). Neuroticism is also related to more concerns but fewer COVID-19 precautions, while conscientiousness is associated with more precautions during pandemics (Aschwanden et al., 2020).

### Leadership

Research on leadership styles suggests that women are more communal, intuitive, sensitive, and empathetic as leaders than men (Rosette and Tost, 2010; Peterson and Bartels, 2017). A meta-analysis found that women tend to exhibit a transformational leadership style which is more relationship-oriented, whereas men tend to show a transactional leadership style which is more task-oriented (Eagly et al., 2003). Men’s leadership is characterized by waiting for problems before innovating solutions, which is consistent with waiting until disaster exacerbates before implementing relief measures (Windsor et al., 2020). Men, on average, tend to prefer having power (defined as control over valued resources) and being feared, while women tend to prefer status (defined as the extent to which one is respected by others) and being loved (Hays, 2013). Female leaders were reportedly rated as less feared than female non-leaders in a forager-horticulturalist population (Garfield and Hagen, 2020). Female leaders are also more likely to navigate social situations successfully and to adapt their behavior accordingly, whilst male leaders, on average, may have a higher likelihood of inflexibly “staying the course” regardless of contextual cues (Peterson and Bartels, 2017). In a pandemic situation, such inflexibility may be catastrophic, as contextual cues from scientists, as well as learning about the outcomes of the pandemic in other countries, can clearly show that inaction—failing to impose measures to stop the virus from spreading—can have worse consequences than imposing societal policies designed to curb the spread of the virus (Haug et al., 2020). On the other hand, the full economic consequences of lockdown policies are also yet to be determined, and may in some cases offset some of the immediate benefits that such policies accrue to population health.

### Competitiveness

Sex differences in competitiveness have been consistently documented in children and adults. Men tend to be more competitive than women across a range of tasks both in large-scale post-industrial societies and in hunter-gatherers (Grainger and Dunbar, 2009; Frick, 2011; Apicella and Dreber, 2015; Hone and McCullough, 2015; Martin, 2020). Studies on competitiveness involving negotiation and bargaining—conducted using laboratory measures of dyadic interactions between North American students—have reported no sex differences in competitiveness, possibly because such contexts are very different from the concept of competition in an evolutionary sense, and from real-life competitiveness for status/power and for attracting a sexual partner (Archer, 2019). Real-life conversations between two males, however, involved much more competitive communication, both verbal and non-verbal, than those between two females, as reported in a study in the United Kingdom (Grainger and Dunbar, 2009). Women are, on average, less willing than men to enter competitive situations, partially because women may be less capable than men in some competitive environments, especially when competing against the opposite sex (Hessami and da Fonseca, 2020; see also Archer, 2019). Even when men and women have similar abilities, men still prefer competition at a much higher rate than women (Hessami and da Fonseca, 2020). A study on the Hadza hunter-gatherers in Tanzania reported that men’s higher competitiveness manifests particularly in male-centric and neutral tasks, whereas in female-centric tasks there is no sex difference in competitiveness (Apicella and Dreber, 2015). Another study conducted in Spain reported that when there was status ranking in a competitive cognitive task, men significantly increased their competitiveness and performance and women significantly decreased their competitiveness; in the absence of status ranking, however, there were no sex differences in competitiveness or performance (Schram et al., 2019). In contrast, when competition is not for money but directly benefits the participants’ children, sex differences in competitiveness disappear, as observed in a study in China (Cassar et al., 2016). A study on children and adolescents from a lower socio-economic segment of Turkey reported that in childhood, there was no significant sex difference in willingness to be a group leader; however, in adolescence, girls became less willing than boys to take on leadership roles, partially because girls had lower self-confidence and social confidence (Alan et al., 2020). One psychological mechanism associated with these sex differences is that girls experience greater competition-induced discomfort than boys in competitive situations, even when competing with same-sex peers (Benenson et al., 2002).

### Risk-Taking

Female leaders’ initial success in tackling the pandemic may be caused in part by women’s greater risk aversion and men’s greater risk-taking (Archer, 2019; Garikipati and Kambhampati, 2020). Men, on average, tend to score higher than women in risk-taking tasks (*d* = 0.49), while women, on average, score higher than men in harm avoidance (*d* = –0.33) (Archer, 2019; see also Ertac and Gurdal, 2012; Gong and Yang, 2012). A study on Israeli executives’ leadership orientations reported that women demonstrated better crisis preparedness by adopting a more holistic approach toward handling crises (Mano-Negrin and Sheaffer, 2004). Similar findings have been reported from hunter-gatherers to bank CEOs. Tanzanian hunter-gatherer males take more risks than females, even as early as in late childhood (Apicella et al., 2017). An analysis of the leadership of S&P 500 firms (*n* = 391) found that firms with female chief financial officers were associated with income-decreasing discretionary accruals, which is in line with sex differences in financial conservatism, risk-aversion, and managerial opportunism (Peni and Vähämaa, 2010). Similarly, a study on 6,971 American commercial banks reported that banks with female CEOs and board chairs were associated with better lending performance and lower default risk in the aftermath of severe real estate price shocks relative to male-led banks, suggesting that female leadership may lead to less risky corporate outcomes (Palvia et al., 2020). These findings are corroborated by a study on Norwegian firms, which reported that introducing gender-balancing quotas that increased women’s representation as firm directors significantly reduced firm risk, though it adversely affected the performance of firms (Yang et al., 2019). A study on company team leaders from the US reported lower risk-taking in female team-leaders relative to males, while innovation scores were lower in female-led teams regardless of the team members’ sex (Zuraik et al., 2020). A meta-analysis has shown that sex differences exist in virtually every area in which risk has been studied, with males engaging in more risk-taking than females (Byrnes et al., 1999). A Swiss study reported that male risk-taking was higher than baseline risk-taking in men in the presence of a male social partner (*d* = 0.87) but not in the presence of a child or a female. Women’s risk-taking was uninfluenced by the presence of other adult males or females; however, in the presence of a baby, women’s risk-taking was substantially lower (*d* = –0.71) from their non-social baseline (Fischer and Hills, 2012). These findings suggest a degree of sex-specific context-sensitivity in men’s and women’s risk-taking, with men’s risk-taking increased by the presence of another man and women’s risk-taking decreased by the presence of a child.

Nevertheless, even when female leaders minimize risks of human suffering by imposing stricter policy measures, such as nation-wide lockdowns, such decisions inevitably lead to greater *short-term* economic risk-taking relative to male leaders (Garikipati and Kambhampati, 2020). Since women’s risk aversion is related to reducing risk of physical harm to themselves and their family and friends (Geary, 2010), and since men are more focused than women on status-seeking (Geary, 2010; Sweet-Cushman, 2016; Archer, 2019; Benenson and Abadzi, 2020), men may be more likely to prioritize immediate economic goals over attempts to minimize health-related risks to others^7^. Furthermore, because women, on average, are more people-oriented, while men, on average, tend to be more things-oriented—and because women have higher empathizing cognitive styles than men (Greenberg et al., 2018; Archer, 2019; Luoto, 2020)—the risks that female leaders view with human suffering may be more salient for them than the risks that female leaders associate with the economy^8^. Economy is more removed from direct human experience and, as an abstract high-level rule-based system, may thus be cognitively more prominent to male leaders, on average, because of men’s higher systemizing cognitive styles (Chari and Goldsmith-Pinkham, 2017; Greenberg et al., 2018; Archer, 2019; Bosquet et al., 2019; Luoto, 2020). Increasing the representation of women in policymaking bodies does not appear to change overall public expenditure; however, higher representation of women in local councils accelerates the expansion of public child care provision and leads to more frequent council discussions on child care (Hessami and da Fonseca, 2020). Furthermore, as men tend to orient toward economic conservatism and women tend to be economically more progressive (i.e., to support policies aimed at equalizing wealth) (Pratto et al., 1997; Harteveld et al., 2019; Hessami and da Fonseca, 2020), it is possible that male political leaders are more concerned about maintaining the economic *status quo* than female leaders. This tendency could result in male leaders being less likely than females to impose lockdowns which restrict economic activity.

### Behavioral Responses to Psychosocial Stress

This view is further supported by sex differences in behavioral responses to psychosocial stress. When experiencing acute psycho-physiological stress, women are more likely to show cooperative behavior which is consistent with the ‘tend and befriend’ hypothesis, while men are more likely to become selfish and competitive, thus showing signs of the ‘fight or flight’ response (Nickels et al., 2017; see also Youssef et al., 2018). More specifically, when exposed to psychosocial stress, males’ tendency to cooperate either did not change or decreased (Nickels et al., 2017; Youssef et al., 2018). Stressed males made lower monetary offers than control men to their partners and tended to behave less prosocially in a risky and potentially dangerous situation, which involved a person in need of help (Nickels et al., 2017). Stressed women, in contrast, offered higher monetary amounts in an economic game and behaved more cooperatively in Prisoner’s Dilemma game compared with control women (Nickels et al., 2017). As with sex differences in pandemic leadership, these results showed a ‘tend and befriend’ response in stressed females as they became more other-oriented, more generous, and more cooperative, while the behavior of males exposed to stress showed signs of the ‘fight or flight’ response.

### Dark Triad and Light Triad

Antisocial personality traits known as the Dark Triad traits (Machiavellianism, narcissism, and psychopathy) may also be highly relevant in a pandemic context, which calls for coordinated, cooperative, and unselfish action. Machiavellianism is associated with manipulative and exploitative behaviors, self-interest, and a ruthless lack of morality; narcissism is characterized by a sense of grandiosity, egotism, and self-orientation; and psychopathy entails antisocial behavior, impulsivity, and a lack of empathy and remorse (Koehn et al., 2018). Men have slightly higher scores on the Dark Triad personality traits than women: cross-national research has revealed small (Cohen’s *d* ≈ 0.20) to large (*d* ≈ 0.70) sex differences in the Dark Triad traits, though the effects are primarily driven by men’s higher psychopathy relative to women (Jonason et al., 2013, 2017; Muris et al., 2017). The Dark Triad traits are positively correlated with dominant leadership, ruthless self-advancement, and prejudice, and negatively correlated with coalition-building (Semenyna and Honey, 2015; Koehn et al., 2018). Psychopathy is also negatively associated with parental investment (Valentova et al., 2020). In the context of the COVID-19 pandemic, individuals with higher Dark Triad traits were less likely to comply with the pandemic restrictions (Zajenkowski et al., 2020) and exhibited less prevention and more hoarding (Nowak et al., 2020).

Kaufman et al. (2019) sought to conceptualize whether there is a complementary set of attributes besides the Dark Triad traits that predicts prosocial rather than antisocial outcomes. The factor-analytically derived Light Traits measure loving and beneficent orientation toward others. The Light Triad consists of three facets: Kantianism (treating people as ends unto themselves), Humanism (valuing the dignity and worth of each individual), and Faith in Humanity (believing in the fundamental goodness of humans). Females had lower scores on the Dark Triad traits (*r* = –0.28) than males, while the Light Triad traits were more common in females than in males (*r* = 0.20). These correlations remained robust even after controlling for agreeableness (Kaufman et al., 2019), but they await replication in other samples as the Light Triad is a more recent addition to the sex difference literature than Dark Triad. Overall, sex differences in Light Triad and Dark Triad traits may influence the extent to which male leaders fail to minimize direct human suffering caused by the pandemic.

### Pathogen Disgust, Health Concern, and Health Behaviors

Importantly for decision-making in a pandemic context, women have higher pathogen disgust than men both generally (Al-Shawaf et al., 2018) as well as in the COVID-19 context (Stevenson et al., 2021), suggesting that women’s decision-making may seek to minimize the spread of a deadly virus more than men’s. The emotion of disgust has far-reaching implications for several areas of psychology, from cognition, judgment, decision-making, and social relationships to health and other behaviors (Al-Shawaf et al., 2018), and so it may be reflected in the decisions that women make even at relatively high levels of abstraction when faced with a pathogenic threat. During the COVID-19 pandemic, women in the general public showed more concern about their own and others’ health (Prichard and Christman, 2020), wearing masks 1.5× more frequently than men (Haischer et al., 2020), even though COVID-19 disease severity and mortality are higher in men (Krams et al., 2020). These sex differences extend even to dreams during the COVID-19 pandemic: a cross-national study on 1,998 women and 890 men reported that women showed significantly lower positive emotions in their dreams and higher rates of negative emotions, anxiety, sadness, anger, body content, and references to biological processes, health, and death than men (Barrett, 2020).

Women also expressed more concern about the financial wellbeing of others than men did (Prichard and Christman, 2020). Survey data from eight countries indicated that women were more likely than men to perceive COVID-19 as a very serious health problem, to agree with restraining public policy measures, and to comply with them (Galasso et al., 2020). A study including 101,005 participants from 55 countries showed that men were more likely to take the risk of going outdoors and were less likely to shelter-in-place than women during the early stages of the pandemics (Götz et al., 2020). A study conducted mainly on Russian participants during the COVID-19 pandemic reported that women had a higher level of anxiety and lower level of spatial mobility than men, suggesting that women take fewer risks by minimizing their mobility during the pandemic (Semenova et al., 2021). A meta-analysis of 85 studies on sex differences in protective behaviors in response to respiratory epidemics and pandemics pre-COVID-19 showed that women were 50% more likely than men to adopt/practice non-pharmaceutical behaviors, such as hand washing, face mask use, and avoidance of public transport (Moran and Del Valle, 2016). A study unrelated to the pandemic context reported that across 67 countries, women showed higher dislike for the suffering of others, as well as more concern about physical and spiritual purity and contamination than men (Atari et al., 2020). Moreover, women with obsessive-compulsive disorder present more contamination/cleaning symptoms while male patients present more sexual-religious and aggressive symptoms (Mathis et al., 2011). In the aggregate, these findings provide additional evidence for the way in which women’s higher empathy, pathogen disgust, care orientation, health orientation, risk aversion, and neuroticism manifest in a pandemic context.

## Evolutionary–Developmental Origins of Sex Differences

Complete evolutionary biological explanations of behaviors or traits need to address four levels of analysis—phylogeny, ontogeny, proximate mechanisms, and ultimate function(s). These can be formulated into four questions concerning any feature of an organism. Answers to these “Tinbergen’s four questions” can be synthesized into a common explanatory framework elucidating the evolutionary origins and biological mechanisms underlying behaviors or traits (Tinbergen, 1963; Luoto et al., 2019a). In this section, we briefly provide such a four-level analysis on sex differences in humans.

### Ultimate Functions

Evolution by natural selection is the only known natural process that propels organisms into higher degrees of functional order, or counteracts the unavoidable increase in disorder that would otherwise ensue (Tooby et al., 2003; Tooby, 2020). All functional organization in undomesticated organisms that is greater than could be expected by chance ultimately results from natural selection and therefore needs to be explained with recourse to it (Tooby et al., 2003; Lewis et al., 2017; Buss, 2020; Tooby, 2020). As living beings, humans are also subjected, body and mind, to the same evolutionary processes as other species. Evolution by natural selection therefore enables a deeper understanding of the origins of human behavior, including sex differences (Archer, 2019; Luoto, 2019; Buss, 2020; Tooby, 2020) and leadership (Sweet-Cushman, 2016; Garfield et al., 2019b; Smith et al., 2020; Van Vugt and von Rueden, 2020). Many factors on different levels, from genetics, local ecology, individual development to social history and phylogenesis, may concomitantly influence the degree of sexual differentiation. Although evolutionary theory provides only a part of the explanation for sex differences, that part is fundamental, offers heuristic power, and helps to reorganize factors that otherwise appear disconnected (DeBruine, 2009; Lewis et al., 2017; Archer, 2019; Luoto, 2019; Buss, 2020).

For instance, natural selection is not separate from cultural explanations of behavior (Figure 1), as “cultural” practices, such as sexual division of labor, are not purely cultural but arise partially because of evolutionary selection pressures acting on sexually dimorphic physiology, cognition, and behavior (Janicke et al., 2016; von Rueden et al., 2018; Archer, 2019). A broader empirically grounded and mechanistic picture on the evolution of sex differences can be acquired from cross-species research on the neurodevelopmental mechanisms that drive sexual differentiation of the brain and behavior (Luoto et al., 2019a; Arnold, 2020; Liu et al., 2020; McCarthy, 2020), a matter to which we return in the section titled “Proximate mechanisms and ontogeny”.

Psychobehavioral sex differences ultimately arise from sexual selection, sexual differentiation of the mammalian brain, sexual division of labor, and their interactions (Figure 1). Sexual selection and sex differences in parental investment have shaped status-striving and power-seeking among men more than in women, resulting in (sometimes violent) competition, risky economic pursuits, and men taking on more leadership positions than women, particularly at higher organizational and societal levels (Gottschall, 2008; Vongas and Al Hajj, 2015; Sweet-Cushman, 2016; von Rueden et al., 2018; Garfield et al., 2019b; Luoto, 2019, 2020; Welling and Shackelford, 2019; Van Vugt and von Rueden, 2020). The mammalian pattern of inter-male competition arises partially because fertile females are a limiting resource for male reproduction (i.e., the Darwin–Bateman paradigm: see Fromhage and Jennions, 2016; Janicke et al., 2016; Hoquet, 2020; and Morimoto, 2020 for recent discussions), which generally leads to higher risk-taking and status-seeking in males relative to females (Archer, 2019; Ronay et al., 2020). Women’s higher empathy and people orientation, in contrast, may be driven by an evolutionarily ancient maternal tendency to care for offspring (Panksepp, 1998; Christov-Moore et al., 2014), interacting with a tend-and-befriend response to psychosocial stress (Nickels et al., 2017; Youssef et al., 2018). Nevertheless, it should be noted that intrasexual rivalry exists also in women (Fisher, 2017), particularly in physical attractiveness and romantic contexts (Rantala et al., 2019; Reynolds, 2021). In the workplace, men and women prefer to compete intrasexually rather than intersexually, but women tend to be more hesitant and calculated in their competitive approach than men (Kocum et al., 2017). Finally, among men, financial success and mating competition/success are correlated—in women, they are uncorrelated (Kocum et al., 2017; see also Luoto, 2019).

While some hold the position that socialization into gender roles causes sex differences in humans, this hypothesis is generally not supported when considering the biological, developmental, neuroscientific, and cross-national evidence more broadly (Christov-Moore et al., 2014; Schmitt, 2015; Janicke et al., 2016; Archer, 2019; Del Giudice, 2019; Luoto et al., 2019a; Liu et al., 2020; Stoet and Geary, 2020). In fact, cross-national evidence indicates that in more gender-egalitarian countries, sex differences are of a higher magnitude than in less gender-egalitarian countries, which is the opposite of what the gender role hypothesis would predict (Schmitt et al., 2008; Falk and Hermle, 2018; Atari et al., 2020; Stoet and Geary, 2020; see also Breda et al., 2020)^9^. Furthermore, since evolutionary processes pre-date social conceptualizations of gender roles by several million years, a complete explanation of the interplay between social conceptions of gender roles and evolved biological predispositions would need to account for how evolutionary processes act as precursors to gender roles (Janicke et al., 2016; Sweet-Cushman, 2016; Archer, 2019).

To bridge this evolutionary approach with the COVID-19 context, there is an important evolutionary aspect behind the hypervigilance, anticipatory anxiety, and threat sensitivity associated with women’s higher neuroticism, risk aversion, and fearfulness (Nettle, 2011; Barlow et al., 2014; Archer, 2019). Error Management Theory predicts that when the cost of missing a real threat is greater than seeing an illusion of threat, evolution selects the less costly error (Haselton and Nettle, 2006). In effect, once a person is fearful, less evidence will trigger a threat response—thus, there will be a higher false alarm rate, which protects against the cost of not perceiving a real threat (Tooby and Cosmides, 2008). The strategic shift in thresholds for signal detection experienced by neurotic individuals leads more often to protective false alarms which are essential in dangerous real-life situations, such as the COVID-19 pandemic. Viability selection and sexual selection (Cornwallis and Uller, 2010) might have acted together in selecting for higher threat vigilance in women (i.e., higher neuroticism) given women’s relatively much lower strength and thus lower self-defense abilities (Lassek and Gaulin, 2009; Nettle, 2011). Although environments experienced by the sexes do not differ substantially, the impacts of undetected threats can be higher for women because of their lower strength and higher parental investment, which may partially increase selection pressures for women’s higher neuroticism, anxiety, and risk aversion (Lassek and Gaulin, 2009; Nettle, 2011). This evolutionary reason of more protective false alarms in women might also be behind women’s higher levels of compliance with protective measures (Moran and Del Valle, 2016; Galasso et al., 2020) and behind female leaders’ decision to act more quickly during the pandemic (Garikipati and Kambhampati, 2020; though see Aldrich and Lotito, 2020), potentially saving more lives. This female-typical ‘false alarm’ line of reasoning from Error Management Theory (Haselton and Nettle, 2006) is of crucial importance for public policy-making during pandemics when the threat of the virus can be more effectively curtailed when it is anticipated rather than experienced.

### Phylogeny

To provide a comprehensive evolutionary account on sex differences, it is valuable to take a broader view into mammalian sexual differentiation of brain and behavior (Janicke et al., 2016; Lonsdorf, 2017; Luoto et al., 2019a; Arnold, 2020; Liu et al., 2020). Evidence of overt sex−biased treatment by others (equivalent to what social constructionists think of as socialization into gender roles in humans) is lacking in many species of non-human animals. In the few species that have been studied, little to no difference has been found in behaviors of mothers toward female and male offspring (Lonsdorf, 2017). Nevertheless, such species show sex differences in behavioral development that resemble differences found in infant humans (Christov-Moore et al., 2014; Lonsdorf, 2017; Archer, 2019). These include differences in physical and social development and in species-typical behaviors such as grooming, playing, object manipulation, and extractive foraging (Lonsdorf, 2017). Immature chimpanzee males engaged in more object-oriented play than females (Koops et al., 2015). Newborn rhesus macaque females that were under 5 weeks old and were raised in a controlled postnatal environment looked more at computer-generated faces of other rhesus macaques and engaged in more affiliative behavior with a human caregiver than newborn rhesus macaque males did (Simpson et al., 2016). Likewise in humans: 12-month-old female infants showed a higher relative preference for a moving face over a moving car than males did (*d* = –0.64) (Lutchmaya and Baron-Cohen, 2002). As in humans, vervet and rhesus monkey females played longer with dolls and plush toys, and males played longer with wheeled toys (Christov-Moore et al., 2014). Asian elephant females tend to be more social and gregarious than males, suggesting that females are more affectionate and seek out others and are sought out by others as company (Seltmann et al., 2019). Human and non-human primate females engage in social grooming more often than males do (Lonsdorf, 2017). In both hamsters and humans, females find same-sex social interactions more rewarding than males do. The finding that oxytocin has a similar mechanistic role in social reward processing in a number of species suggests that sociality and sex differences in sociality may have a deep common evolutionary origin (Feng et al., 2015; Hung et al., 2017; Borland et al., 2018).

An analysis of 76 non-human mammal species (Smith et al., 2020) showed that female-biased leadership manifested most often as females leading collective movements. Of the 76 non-human mammal species, female-biased leadership was reported only in eight species: (1) bonobos (*Pan paniscus*), (2) ring-tailed lemurs (*Lemur catta*), (3) black-and-white ruffed lemurs (*Varecia variegata*), (4) killer whales (*Orcinus orca*), (5) spotted hyenas (*Crocuta crocuta*), (6) African lions (*Panthera leo*), (7) African bush elephants (*Loxodonta africana*), and (8) Asian elephants (*Elephas maximus*). Male-biased leadership therefore is the most typical across the mammalian lineage (Smith et al., 2020), including humans, both in large-scale post-industrial societies as well as more egalitarian, small-scale societies (Garfield et al., 2020).

The closest living relatives of modern humans that have female-biased leadership are bonobos. It has been suggested that same-sex sexual behavior has allowed female bonobos to overcome the phylogenetic legacy of male dominance in primates by “making love, not war” (Smith et al., 2020). Female-biased leadership in bonobos is characterized by peaceful social interactions—and it is common for females to use genital contact to reduce tensions with both males and females (Smith et al., 2020). Leadership in bonobos is therefore non-isomorphic in relation to human leadership. Chimpanzee leadership is male-biased and resembles human leadership more than bonobo leadership does; male chimpanzees, for instance, lead in group hunting, within-group interventions, and intergroup warfare (Smith et al., 2020).

### Proximate Mechanisms and Ontogeny

The proximate level of analysis (see e.g., Lewis et al., 2017; Zietsch et al., 2020, for a discussion of the proximate–ultimate distinction) focuses on the biological and/or social mechanisms underlying a trait or behavior. Accumulating evidence indicates that sex hormones play a key role not only in sexual differentiation of the brain (Figure 1; Luoto et al., 2019a,b; Arnold, 2020), but also in sexual dimorphism in the activation of the endocannabinoid and the mesocorticolimbic pathways, both of which create sex differences in reward-seeking behaviors. These sex differences, though operating within a continuum, are central in shaping a number of life outcomes from sexual behavior, sensation-seeking, substance use, and risk-taking to variation in health (Struik et al., 2018; Becker et al., 2019; Luoto et al., 2019a,b, 2021; Mauvais-Jarvis et al., 2020).

During critical periods of development in fetal and neonatal life, testicular secretions have permanent effects on the brain, driving sexual differentiation of the brain (Arnold, 2017; Forger, 2018; Kret and De Gelder, 2012; Luoto et al., 2019a,b). There are three major classes of proximate sex-biasing factors: sex chromosome effects (the differential action of X and Y genes or chromatin that are out of balance in XX and XY genomes), and organizational and activational effects of gonadal hormones (Arnold, 2020; see also McCarthy, 2020). Unlike activational effects, the early organizational effects of gonadal hormones are considered irreversible, creating various degrees of masculinized phenotypes in brain, physiology, cognition, and behavior (Figure 1; Luoto et al., 2019a,b; Arnold, 2020).

Exposure to androgens has an effect on neuronal survival and connections (Kret and De Gelder, 2012) and can play an important role in the sex-specific development of the endocannabinoid system, which directs reward-related behavior (Struik et al., 2018; Luoto et al., 2019b) and which may therefore partially underlie the psychological sex differences reported above (Luoto et al., 2019a). Testosterone, for instance, has both organizational and activational effects on risk aversion and choosing risky careers in finance (Sapienza et al., 2009; see also Apicella et al., 2015). Individuals with genetic disorders provide additional evidence on the ways in which sex hormones direct development. Congenital adrenal hyperplasia (CAH) is a genetic disorder that affects adrenal glands and results in an overproduction of testosterone in affected women. A study comparing unaffected men and women in people and things orientation reported a very large (*d* = –2.02) sex difference, with men scoring higher on things orientation and women scoring higher on people orientation (Beltz et al., 2011). Interest in things relative to people was higher in women with CAH than in unaffected women (*d* = 0.75) as can be predicted by the higher dose of testosterone to which CAH women are exposed (Beltz et al., 2011). People and things orientation was correlated with the degree of androgen exposure: women with a severe form of CAH had higher scores on things orientation than women with milder forms of CAH. CAH women also reported a higher interest in scientific occupations (*d* = 0.56) and mechanical occupations (*d* = 0.64) but lower interest in social occupations (*d* = –0.30) than their unaffected siblings (Beltz et al., 2011), which highlights the masculinizing effect of testosterone (cf. Luoto et al., 2019a).

While significant differences between men’s and women’s brains have been reported in adulthood (Del Giudice, 2019), Wheelock et al. (2019) were the first to report the existence of sex differences in the human brain *in utero*. More specifically, Wheelock et al. (2019) reported that functional connectivity of the human brain is organized into highly fragmented prenatal brain networks, and that prenatal functional connectivity varies with regard to fetal sex and gestational age. These findings provide strong evidence against claims about brain sexual differentiation occurring because men and women are differentially socialized into gender roles (Rippon, 2019). Wheelock and colleagues’ findings on the prenatal sexual differentiation of the human brain further reinforce biological theories of brain sexual differentiation and core gender identity development (Fisher et al., 2018; Luoto et al., 2019a; Arnold, 2020; McCarthy, 2020). Neurodevelopmental theories of gender identity development are also supported by longitudinal research. While hormone exposure significantly predicted gender development in girls, their mothers’ socialization efforts to feminize the daughters had negligible effects: women subjected to more testosterone in prenatal development showed masculinized behaviors in adulthood despite their parents’ socialization efforts to make the daughters more feminine (Udry, 2000; see also Luoto et al., 2019a).

Research on the sexual differentiation of the mammalian brain, mirror neurons, theory of mind, and the evolutionary origins of empathy (Christov-Moore et al., 2014; Peterson and Bartels, 2017; Luoto et al., 2019a; Luoto, 2020) suggests that there are biological mechanisms underlying the psychological sex differences reviewed above. Women are better than men at interpreting others’ intentions and actions, demonstrating an improved domain-specific ability to read others’ minds (Ibanez et al., 2013; Varella, 2018). Psychologically, this sex difference is mediated by empathy (Ibanez et al., 2013), a trait in which sex differences are well known (Archer, 2019). Developmentally, theory of mind is affected by prenatal androgen exposure (Khorashad et al., 2018), which is an important neurodevelopmental mechanism giving rise to many psychobehavioral sex differences (Luoto et al., 2019a,b; Arnold, 2020), including people–things orientation (Beltz et al., 2011; Luoto, 2020). Women have increased mirror neuron activity when evaluating the emotions of others (Peterson and Bartels, 2017), and men and women differ qualitatively in how emotional information is integrated to support decision-making processes (Christov-Moore et al., 2014). In the aggregate, these findings suggest that women may be more empathetic leaders than men (cf. Sergent and Stajkovic, 2020), and that the sexual differentiation of the mammalian brain is one of the main underlying biological processes causing psychobehavioral sex differences in humans (Figure 1).

## Sexually Dimorphic Leadership Specialization Hypothesis

We have collated recent evidence which suggests that female leaders may be more effective than male leaders in a pandemic context, particularly reducing mortality outcomes. We connected this leadership strength with women’s evolved sex-typical psychobehavioral traits. Given the stability, universality, and phylogenetic inertia of those sex-typical traits (Geary, 2010; Lonsdorf, 2017; Archer, 2019), it is possible to infer that a similar leadership success of women during disease outbreaks would, for the same sex-typical psychobehavioral characteristics, also have existed during ancestral times, depending also on followers’ reactions, contextual factors, and local cultural norms. After all, humans have an evolved leadership psychology (Garfield et al., 2019b; Van Vugt and von Rueden, 2020), and this sex-specificity could be a part of it.

The hypothesis about the possible ancestral effectiveness of female leadership during a disease outbreak complements the literature on the evolved aspects of male political leadership, particularly regarding the different ecological and sociopolitical threats that societies have faced throughout primate evolution (cf. Watts, 2010; McDonald et al., 2012; Smith et al., 2020). Males, on average, engage in more risk-taking and aggressive activities than women, and those sex differences have a long evolutionary history (Van Vugt, 2009; Geary, 2010; Sweet-Cushman, 2016; Archer, 2019). In recent and in ancestral times, intergroup conflicts were frequent and entailed a substantial mortality rate (Bowles, 2009). Formidable and dominant male community leaders would have been preferred particularly in times of intergroup conflict and war (Hayden et al., 1986; Grabo and van Vugt, 2018; Garfield et al., 2019b), which could have resulted in higher reproductive success for the experienced warrior leaders (von Rueden et al., 2011; Glowacki and Wrangham, 2015; von Rueden and Jaeggi, 2016), despite the individual agency of the male leader being less crucial than social networks in between-group violence (Glowacki et al., 2016). Tigue et al. (2012) found that manipulated lower voice pitch of recordings of US presidents was more strongly associated with physical prowess in a wartime voting scenario and that participants preferred to vote for the candidate with the lower-pitched voice, which indicates dominance (Wolff and Puts, 2010; Aung and Puts, 2020). Similar results have been reported in other studies (Little et al., 2007; Halevy et al., 2012; Spisak et al., 2012). Facial cues associated with perceived height and masculinity in potential leaders’ faces are valued more in a wartime context vs. peacetime context (Spisak et al., 2012; Re et al., 2013; Grabo and van Vugt, 2018). Preference for leader dominance seems to be uniquely driven by the intuitive notion that dominant leaders are better in giving an aggressive response in times of social conflict (Laustsen and Petersen, 2017). Current evidence suggests that the predominant preference for male over female political leaders could be a byproduct of the ancestral preference for physically formidable allies (Murray and Carroll, 2020). Furthermore, other aspects of the dominant male leader can also be relevant to the in-group, such as better coordination, negotiation, and efficiency at suppressing free-riding (Lukaszewski et al., 2016; see also Varella et al., 2021; Yong and Choy, 2021).

This male and female leadership differentiation, preference, and effectiveness could be either (1) a byproduct of more general sex differences in physiology, cognition, and behavior (cf. von Rueden et al., 2018; Archer, 2019), or (2) an evolved sex-specific specialization in different kinds of leadership styles. We refer to this second alternative as the *sexually dimorphic leadership specialization hypothesis*. According to this hypothesis, it would have been more effective to have male community leaders during ancestral (and recent) times of frequent wars, aggression (both intergroup and intragroup), and possibly during geological and other natural hazards, while during disease outbreaks and famines it would have been more effective to have female leaders. This possible sex-specific specialization would result from the coevolution between male and female roles as leaders in which men’s and women’s psychological strengths were recurrently recruited by society and/or followers and used for leadership in different and correspondent threat contexts based on the effectiveness of leadership outcomes in each context. This hypothesis can also be extended to coalitions of leaders; those coalitions with a higher proportion of males would deal better with violent conflicts, while those with a higher proportion of females would deal better with epidemics.

Although there are interrelationships among all classes of environmental threats, they do not always appear simultaneously nor with the same frequency. Infectious disease outbreaks increase ethnocentrism and resource scarcity which later tend to lead to armed conflict and civil wars (Letendre et al., 2010). Conversely, times of war and conflict tend to contribute to pandemic outbreaks (Habicht et al., 2020). However, many more factors trigger conflicts, such as increases in temperature or in extreme rainfall (Hsiang et al., 2013; see also Van Lange et al., 2017), or social/economic inequalities (Stewart et al., 2002), than disease outbreaks, so much so that violent conflicts are much more frequent (Stewart et al., 2002; Letendre et al., 2010; Hsiang et al., 2013) than epidemics and pandemics (Hays, 2005; Habicht et al., 2020). Hence, this disparity creates differential selective pressures on leadership which underliers the sexually dimorphic leadership specialization hypothesis and, consequently, the observed higher prevalence of male leadership.

The premise of this hypothesis is the idea that sexually dimorphic leadership specialization is an *exaptation*, as it hypothetically arose from sexually dimorphic psychological traits which evolved for other purposes, such as higher status-seeking, particularly in high-level organizational contexts, as well as male–male aggression in men—and maternal care, empathizing, and pathogen disgust in women (e.g., Geary, 2010; Archer, 2019). An exaptation is a feature that improves fitness in a way that differs from its “original” evolutionarily selected role, having acquired a novel function in the course of evolution (Gould and Vrba, 1982; Gould, 1991; Buss et al., 1998; Luoto, 2019). A correspondent and consequent new phenomenon stemming from this process of exaptation would be a context-specific preference for leaders of each sex.

The evidence we have reviewed suggests this might be the case with sexually dimorphic leadership specialization, though it would be necessary to establish the kinds of fitness benefits (and costs) that women accrue from positions of leadership (cf. Sweet-Cushman, 2016; Garfield et al., 2020). There is existing research on the fitness benefits of leadership for male leaders, namely more in-pair surviving offspring as well as more extra-marital affairs and higher wife quality (von Rueden et al., 2011; von Rueden and Jaeggi, 2016; Spisak, 2020). A study using ethnographic records from 60 cultures showed that male leaders tend to be more polygynous than non-leaders across cultures (Garfield et al., 2019a).

As such, the sexually dimorphic leadership specialization hypothesis is consistent with how sex differences in parental investment and mating competition coevolve with parental care specialization, based partially on ecological factors (Henshaw et al., 2019). Evolutionarily, parental investment consists of two or more distinct activities: provisioning and defense. Consequently, parents may care more efficiently if they specialize in a subset of these activities when it is inefficient for a single parent to provide multiple types of care (Henshaw et al., 2019). This kind of parental care specialization occurs in many taxa (Janicke et al., 2016; Henshaw et al., 2019). Based on what is known on psychobehavioral sex differences and their evolution in humans, the sexually dimorphic leadership specialization hypothesis extends (Figure 1) models on the evolution of parental care specialization (Trivers, 1972; Janicke et al., 2016; Henshaw et al., 2019)—and the biological constraints of parental care on economic activity (Starkweather et al., 2020)—to leadership types.

Nevertheless, more work is required to address the question of fitness benefits and costs of leadership in women. It is highly likely that men and women differ with regard to the fitness-related benefits and costs associated with positions of leadership—and that this difference is caused and/or mediated by sex differences in (1) parental investment, (2) age-related fertility decline, (3) mate preferences, (4) reproductive physiology, (5) reproductive ecology, and (6) sexual and reproductive decision-making (Trivers, 1972; Valeggia and Núñez-de la Mora, 2015; Sweet-Cushman, 2016; García et al., 2018; Archer, 2019; Buss and Schmitt, 2019; Luoto, 2019; Hughes et al., 2021). Evolutionary theory supports the view that men are able to derive significant reproductive benefits from politically ambitious behavior, while fewer benefits accrue to women from similar behaviors (Sweet-Cushman, 2016; see also von Rueden et al., 2011; Buss and Schmitt, 2019; Garfield et al., 2020). Women who try to use resources and status to attract multiple mates are not distinctly favored by natural selection, whereas men are (Geary, 2010; von Rueden et al., 2011; Sweet-Cushman, 2016; Luoto, 2019)^10^. However, politically influential women may be able to bear healthier offspring (Alami et al., 2020), possibly because of higher resource availability which supports somatic and immunological development (cf. Krams et al., 2019; Rubika et al., 2020). Moreover, in a hunter-gatherer society, male and female leaders share a similar phenotypic profile and are rated as having higher spouse quality than non-leaders; thus, they tend to be married to one another (von Rueden et al., 2018; Garfield and Hagen, 2020), which might improve offspring quality and social status^11^.

An evolutionary approach to leadership recognizes that ancestrally there may have been limited incentive for women to take the risks associated with gaining and holding on to power in the public sphere, which partially explains sex differences in leadership prevalence and political ambitions (Sweet-Cushman, 2016; cf. Garfield et al., 2020; Smith et al., 2020). Nevertheless, it is possible that if leadership is analyzed on different levels of social organization (e.g., within and between families), men and women could show different leadership pattern on different levels—women up to the extended family level, men at higher organizational and societal levels—to the extent that if taking family leadership into account, the overall sex difference in leadership could diminish, vanish, or even reverse, favoring females (Garfield et al., 2019a). Cross-nationally, men’s status hinges more on athleticism, bravery, physical formidability, hunting skills, and aspects of leadership, while women’s status is more dependent on physical attractiveness and domestic skills (e.g., processing food, childcare) (Buss et al., 2020). Female leaders in horticultural and hunter-gatherer societies were more likely than male leaders to be in a polygynous marriage with a high-quality spouse, to receive *more* social, reproductive, and material success whilst having *less* prosocial competence than male leaders (Garfield et al., 2020). These surprising results suggest that female leaders tend to be high-status wives who gain social influence across the lifespan through their high-quality polygynous spouse, extended kin, and social networks (Garfield et al., 2020); however, because of the exploratory nature of this study, as well as the small sample size of female leaders, these findings await further confirmation.

Notably, the sexually dimorphic leadership specialization hypothesis does not suggest that effective leadership is exclusive to either males or females, nor that half of the time each sex would be in charge as a leader; rather, it posits that, on average, evolved predispositions would bias men’s and women’s leadership styles to focus relatively more on different areas (intergroup aggression vs. health and societal care) which become prominent during different contexts.

## Assessing the Evidence for the Sexually Dimorphic Leadership Specialization Hypothesis

Despite the lack of direct systematic evidence on the sexually dimorphic leadership specialization hypothesis, it is supported by circumstantial evidence stemming from diverse sources from hunter-gatherers to large-scale post-industrial societies, which we touched on above and review in more detail below. We should in any case note that the fact that female leadership is phylogenetically far less prevalent than male leadership and shows phylogenetic inertia in the mammalian lineage (Smith et al., 2020) could be tentatively interpreted as evidence against the sexually dimorphic leadership specialization hypothesis, seeing that the kinds of contexts which the hypothesis posits will select for female leadership (disease outbreaks, famines) were sporadic but recurring threats both evolutionarily and in recent history. We therefore do not rule out the hypothesis that sex differences in leadership are merely a coincidental byproduct of more general psychological sex differences which evolved for purposes other than leadership.

Before we review evidence for the sexually dimorphic leadership specialization hypothesis, we also note that there is some evidence against it. A study on sex differences in state leadership in Europe between 1480 and 1913 reported that queens engaged more in wars in which their polity was the aggressor than kings did (Dube and Harish, 2020). However, this effect varied by marital status. Unmarried queens were attacked more than kings. Among married monarchs, queens acted as attackers more than kings. The results suggest that unmarried queens may have been attacked because they were perceived to be weak, while married queens may have had greater capacity to attack supported by their spouses who helped them rule (Dube and Harish, 2020). Furthermore, if queens tended to lose wars more than kings, it would provide evidence in favor of the sexually dimorphic leadership specialization hypothesis, which posits that males are more effective leaders in a wartime context. Evidence at this high level may be subject to complex modifiers, which is why evidence of effectiveness of female leaders during war vs. pandemics may be propitiously analyzed at smaller social scales.

Given the stability, universality, and phylogenetic continuity of the relevant sex-typical traits (Geary, 2010; Lonsdorf, 2017; Falk and Hermle, 2018; Archer, 2019) in which sex-differences in leadership are presumably based (Sweet-Cushman, 2016; Garfield et al., 2019b; Smith et al., 2020), convergent evidence from hunter-gatherers and large-scale post-industrial societies tends to support the sexually dimorphic leadership specialization hypothesis. However, besides the current pandemic, there is a lack of systematic evidence related to sex-dependent effectiveness of leadership during public health crises (cf. Knebel et al., 2012). There are some selected historical cases that arguably could point to where future systematic studies could be conducted to test the hypothesis. There are historical examples of female Native American leaders who saved lives by connecting tribal affairs and public health programs against contagious diseases, such as tuberculosis (Trennert, 1998; Davies, 2001). Of all indigenous female roles, few are as notable as the medicine woman/traditional healer (Lajimodiere, 2013; Mji, 2019). There are scattered historical examples of women nurses providing significant leadership in healthcare crisis response (Schoch-Spana, 2001; Bristow, 2012; Knebel et al., 2012; Patterson, 2012; Fawole et al., 2016), although there are also some instances of male nurse leadership (Evans, 2004). During the foot-and-mouth disease and the bovine spongiform encephalopathy crisis (1990s–2000s), there was a contrast between the disorganized and slow UK response led by males and the rapid and effective French response led mostly by females (Kahn, 2020). Moreover, women have led initiatives developing response, relief, and recovery measures from many past disasters, such as hurricanes and disease outbreaks (Enarson, 2012, p. 245). We do not claim that these instances are an extensive literature review nor that they systematically test the sexually dimorphic leadership specialization hypothesis, only that together they can offer an initial and possible pattern in that direction, which should guide future systematic analyses on sex differences in leadership during disease outbreaks.

Neuroscientific evidence points to distinct and antagonistic brain areas related to two leadership roles: the task-oriented leadership role is attributed to activation of the task-positive network, while the socio-emotionally oriented leadership role relies more on the default mode network (Boyatzis et al., 2014). These brain specializations and mutual suppression of activities related to different leadership styles might be the neurobiological basis for sexually dimorphic specialization in leadership. There are even genetic specificities of each leadership style: additive heritability (the effect of multiple genes that exert influence in a linear or additive fashion) is more related to transactional leadership style, while non-additive heritability (interactive effects of different alleles: within-locus dominance and across-locus epistasis) is more related to transformational leadership style (Johnson et al., 1998). Importantly, these leadership roles show a sex difference (Peterson and Bartels, 2017). The task-oriented role of leadership is related to the inflexible “staying the course” of male leadership style and its autocratic dimension, while the socio-emotionally oriented role matches the more intuitive, sensitive, empathetic, and democratic leadership styles of women (Eagly and Johnson, 1990; Peterson and Bartels, 2017). Moreover, men, on average, tend to prefer power, resources, and being feared, while women tend to prefer status, being respected, and loved (Hays, 2013). Such overall patterns in leadership are consistent with the sex-typical psychobehavioral strengths of women with regard to empathy, people orientation, care and health orientation, emotional expression, and sense of fairness and purity—and of men with regard to risk-taking, competitiveness, systemizing, the Dark Triad traits, physical aggression, violence, pain tolerance, and lack of fearfulness, shame, and guilt (Geary, 2010; Varella et al., 2016; Archer, 2019; Atari et al., 2020; Luoto, 2020; Prichard and Christman, 2020).

Evidence from occupational choices shows that homemaking (94% women), administration (75%), and healthcare (70%) are the top three careers with high proportion of women—and importantly, those occupations require the highest empathizing-biased cognitive style (Manning et al., 2010) as well as people orientation (Tay et al., 2019). In contrast, professions such as general management and government/military (both 64% men) and business development (62% men) favor individuals with higher systemizing cognitive styles (Manning et al., 2010; see also Luoto, 2020). Similar patterns are found in academic publishing. Nursing and health professions favor empathizing cognitive styles and a strong people orientation, and they have a high degree of female researchers/authors. Academic fields with strong systemizing requirements and a high things orientation, including economics, tend to have a much higher proportion of male researchers as authors (Luoto, 2020). A study on 22 established democracies between 1970 and 2000 reported that an increased proportion of women in the legislature decreased defense spending and conflict behavior, even after controlling for government partisanship and the rights of women in society (Koch and Fulton, 2011). Other research on policymaking has reported significant sex differences in implementing policies related to health, development aid, the environment, defense spending, women’s issues, and welfare policy (Hessami and da Fonseca, 2020). The evolved sex-typical psychobehavioral strengths may lead to these distinctions of policymaking and vocational choice, whilst also predisposing leaders to use their talents and strengths in the respective leadership contexts predicted by the sexually dimorphic leadership specialization hypothesis: women focusing more on healthcare, welfare, and society, and men focusing more on intergroup aggression, military, and the economy.

The hunter-gatherer socio-ecological way of life resembles the social structure and functioning of ancestral human lifestyles during the Pleistocene, and is thus informative with regard to *Homo sapiens* evolutionary history (e.g., Sweet-Cushman, 2016; though see Moreau, 2020). Male leaders across 59 mostly non-industrial populations had higher military command and distributed resources more often than female leaders did (Supplementary Figure S12 in Garfield et al., 2020). Anthropological evidence from egalitarian small-scale societies suggests that leadership emerges facultatively according to context-specific demands in serving the collective interests rather than from a single powerful authoritative figure (Garfield et al., 2019b). Human leaders tend to lead in one or a few domains, and there are usually many concomitant leaders in different areas such as hunting, group defense, and traditional healing (e.g., shamans) (Garfield et al., 2019b). It is probable that humankind’s earliest politicians, headmen, were exclusively men (Sweet-Cushman, 2016). Although shamans and traditional healers can be either male or female and the empirical evidence is ambiguous about it in small-scale societies (e.g., Brown et al., 2006; Jaradat and Zaid, 2019; Audet et al., 2020), the healing practices of shamans (Garfield et al., 2020), particularly involving trance performances of ‘spirit’ possession, are often done by women in larger and more hierarchically layered societies (Wood and Stockly, 2018). There are even cases in which males change their gender roles by dressing and behaving in feminine ways to be able to practice shamanism (Tomášková, 2013). At least 10% of non-industrial societies have women in leadership positions, and in some instances shamans are also considered leaders (Garfield et al., 2019b).

An evolutionary view of leadership across species and societies has identified two main widespread types of leadership: one based on physical and social formidability (dominance), and another based on information and skills (prestige) (Garfield et al., 2019b; Van Vugt and von Rueden, 2020). This framework is consistent with empirical evidence showing that there are two distinct and viable routes to ascend in social rank: dominance (use of force and intimidation to induce fear, and selfishly manipulating the group resources) and prestige (sharing of expertise/valued knowledge or know-how to gain respect) (cf. Cheng et al., 2013; Maner and Case, 2016). Although the same leader can make use of both types of strategies, the evolved sex-typical psychobehavioral tendencies influencing leadership may incline male leaders to rely on the dominance strategy more often (cf. Evans, 2004) and female leaders to more frequently use the prestige strategy (cf. Holmgren et al., 2019). Indeed, female leaders in a forager-horticulturalists society in Ethiopia showed high prestige but low dominance, whereas male leaders were high on both prestige and dominance (Garfield and Hagen, 2020). We predict that this sexual dimorphism in leadership styles becomes more accentuated under distinct threat contexts (e.g., intergroup conflict vs. disease outbreaks). Such sex-specific responses to threats would be in line with the female-typical ‘tend-and-befriend’ response and the male-typical ‘fight or flight’ response to psycho-physiological stress (cf. Nickels et al., 2017).

Organizational literature on modern company leaders also points in the same direction as the above evidence. Women tend to be mostly chosen to lead whenever an organizational crisis is minimal to moderate and stems primarily from within the organization, while men tend to be chosen as leaders whenever the crisis threatens the very existence of the organization and its source is an external threat (Vongas and Al Hajj, 2015). Although within-group threats such as free-riding and crimes from other group members also increase preference for dominant-looking leaders (Bøggild and Laustsen, 2016) or those described verbally as dominant (Zhu et al., 2021), female leaders are preferred for the resolution of within-group disputes while male leaders are preferred to lead under conditions of intergroup conflict (Van Vugt and Spisak, 2008). As any microscopic pathogenic agent enters the group and slowly contaminates in-group members, it constitutes a within-group crisis. Hence, according to this literature, it is more probable that women would be assigned to lead the group out of this kind of pathogen-induced threat, in accordance with the sexually dimorphic leadership specialization hypothesis. The hypothesis is also consistent (though not fully overlapping) with evidence indicating that human and non-human animal leaders are often chosen based not necessarily on sex, but on the attributes that signal their competence to lead group activities (Smith et al., 2020; see also Garfield et al., 2020).

By introducing this hypothesis, we aim to highlight this pattern of sex specialization in leadership and point to possible avenues for future research. The sexually dimorphic leadership specialization hypothesis, which posits that the balance of male/female leadership shifts depending on the context of the main threat to the group, is not offered as a mere ‘just so story’ (cf. Varella et al., 2013). Instead, we have provided deeper insights based on the patterns observed in existing literature from various fields, and invite further testing by offering convergent circumstantial evidence for the hypothesis. These future studies would thus go beyond the ‘null hypothesis’ of seeing women’s leadership success during the COVID-19 pandemic merely as a recent byproduct of evolved sex differences, which only now happen to manifest in a leadership context. The sexually dimorphic leadership specialization hypothesis could be further tested by studying the sex-specific fitness benefits and costs associated with leadership (cf. Garfield et al., 2020; Spisak, 2020), as well as details on how a population’s socioecological and cultural contexts influence the type of preferred leader.

Based on the sexually dimorphic leadership specialization hypothesis, it can be predicted that women, feminine individuals, or female-biased or feminine coalitions would be more motivated to help save lives during disease outbreaks, leading more effective societal responses, particularly in less patriarchal, more gender-egalitarian societies where women have unobstructed access to the political sphere. In small-scale societies, anthropologists can study sex differences in leadership during disease outbreaks, while historians are encouraged to focus on sex differences in formal (elected) or informal (e.g., head nurses) leadership during past disease outbreaks. In lab experiments, participants primed with pandemic (versus war-time) contexts are predicted to positively evaluate, vote for, or trust in feminine (versus masculine) political candidate faces/voices. Both manifest protective/caretaking behaviors during disease outbreaks and psychological tendencies/bias toward protection/caretaking should be empirically assessed in studies on female vs. male leaders.

## Discussion

Evolutionary science has been applied to understanding and predicting specific outcomes of the COVID-19 pandemic in various ways (Arnot et al., 2020; Corpuz et al., 2020; Seitz et al., 2020; Varella et al., 2021). However, sex differences in pandemic leadership have not been previously approached from an evolutionary perspective. As such, an evolutionary approach offers an alternative explanation to other hypotheses on sex differences in leadership and policymaking. In fact, a prominent theoretical position in the political economy literature suggests that personal characteristics of officeholders do not matter for policy choices, yet empirical evidence reviewed here and elsewhere does not support this hypothesis (Hessami and da Fonseca, 2020).

A convergence of key findings strengthens the case for an evolutionary approach to leadership in general, and manifest sex differences in leadership behaviors in particular. Leadership is universal among industrial and small-scale societies, including hunter-gatherers (Zagorsek, 2004; Price and Van Vugt, 2014; Garfield et al., 2019b). Possible universal traits of leaders include qualities such as being knowledgeable, intelligent, and capable in conflict resolution (Garfield et al., 2020). There are clear shared phylogenetic (among big carnivores, great apes, and extinct hominids) and ontogenetic (among children, adolescents, and adults) patterns of leadership (Garfield et al., 2019b). Propensity for leadership is heritable, with an estimated genetic contribution of 44% in women and 37% in men (Chaturvedi et al., 2012). A specific genotype is associated with the tendency to occupy a leadership position (De Neve et al., 2013). There are specific neural networks underlying differentiated leadership types (Boyatzis et al., 2014; Peterson and Bartels, 2017), and a specific set of cognitive skills utilized in leadership (Mumford et al., 2017). There are sex differences in leadership styles (Peterson and Bartels, 2017; Garfield et al., 2019b), and evidence for differential reproduction in male leaders of small scale hunter-gatherer societies (e.g., polygyny among leaders and monogamy among followers) (von Rueden et al., 2011; Garfield et al., 2019b), which suggests that sexual selection drives these differences. Leadership has the important evolutionary and social function of instrumentally solving collective action dilemmas while balancing the interests of leaders and followers according to reciprocal altruism and kin selection (von Rueden et al., 2014). All this points to the possible evolved status of the tendency toward leadership in humans: an evolved leadership psychology (Van Vugt and Kurzban, 2011; Sweet-Cushman, 2016; Garfield et al., 2019b; Van Vugt and von Rueden, 2020).

In light of the individual variation within each sex, which tends to be larger than variation between the sexes (e.g., Archer, 2019; Del Giudice, 2019; Landry et al., 2019; Luoto et al., 2019a,b), future studies should analyze whether there are intrasexual differences on masculinity–femininity continuum that mirror the sexually dimorphic tendency in leadership efficacy. After all, cues of masculinity–femininity can be more influential than actual sex cues at predicting perceptions of leadership (Spisak et al., 2012). In this light, our review and hypothesis can be better understood in a more nuanced fashion and focused on maleness and femaleness rather than simply presenting a male vs. female dichotomy (cf. the phenotypic continua in Figure 1).

## Limitations

This review has some limitations, as there is still a shortage of empirical studies on many fronts, particularly in a pandemic leadership context. One obvious area for further study would be to analyze political leaders’ personality traits, particularly with regard to the psychological sex differences reviewed in this article, using the general population as a reference sample (cf. Wille et al., 2018). Furthermore, some female leaders, such as Jacinda Ardern of New Zealand, have also been praised for their communication skills, which is consistent with the general pattern of higher verbal skills and language ability in women relative to men (Archer, 2019); however, few studies have been conducted on sex differences in communication and language use in a pandemic leadership context (though see Sergent and Stajkovic, 2020; Dada et al., 2021). Another limitation inherent in an evolutionary approach to leadership is the challenge of studying patterns of leadership in extinct hominin species because relevant findings cannot be extracted from fossil records alone, beyond what is possible to infer using body size sexual dimorphism. The fact that we have stressed biological, evolutionary, and mostly dispositional psychological facets does not exclude the possibility that other factors, some of which are contextual or cultural traditions (cf. Hewlett and Hewlett, 2007), might also contribute to female leaders’ success during the pandemic, such as reliance on scientific recommendations, consistent public communication about the safety measures, emphasis on uniting the country, the composition of the entire political team, the dominant political ideology of the country, and the leader’s educational, personal, and political backgrounds, among others (e.g., Luoto, 2020; Stoet and Geary, 2020).

For instance, to the extent that female politicians are chosen relatively more often to represent liberal political parties and have more liberal values themselves (e.g., Pratto et al., 1997; Oniszczenko et al., 2011; Harteveld et al., 2019), their decisions may reflect liberal values such as equality, social change, and system reform, rather than conservative hierarchic economic values (cf. Oniszczenko et al., 2011; Claessens et al., 2020; Hessami and da Fonseca, 2020). Therefore, studies on sex differences in pandemic leadership should analyze the extent to which political party affiliation mediates the relationship between leaders’ sex, the policies they implement, and pandemic-related outcomes. We note that the two national-level studies reviewed in this article did not analyze how leaders’ political party affiliations might act as a mediating variable (Garikipati and Kambhampati, 2020; Purkayastha et al., 2020), while the state-level study used political affiliation as a control variable (Sergent and Stajkovic, 2020). We suggest that rather than treating political affiliation as a “nuisance” variable that needs to be controlled for, it might be better conceptualized as a statistical (and theoretical) mediator (cf. Harteveld et al., 2019; Luoto and Jonason, 2019).

Moreover, Garikipati and Kambhampati’s (2020) comparison between women- and men-led countries was done without differentiating whether each female leader was a governing leader (such as a prime minister: head of government) or serving in more of a titular role (such as a president: head of state). This analytical decision yields higher statistical power but may obfuscate some of the results based on who were the most influential decision-makers behind pandemic policies (cf. Baekkeskov and Rubin, 2014), with titular leaders having potentially less direct influence on pandemic policy-making than governing leaders.

More generally, the non-randomized assignment of women to political positions constitutes a complex empirical challenge (Hessami and da Fonseca, 2020; Windsor et al., 2020), which is why a multidisciplinary broad-perspective approach, as applied in this article, can best address the complexities of observed sex differences in leadership behaviors and their outcomes. A related potential limitation is that executive positions can have a homogenizing effect on personality and that psychologically more male-typical women may be more likely to pursue and to be chosen for leadership positions (Wille et al., 2018). This may lead to range restriction, a process in which the subjects of a sample are (directly or indirectly) selected from the original population on the basis of their idiosyncratic personal characteristics and therefore do not represent a random sampling of the population (Del Giudice, 2019). It may therefore not be possible to directly extrapolate these findings on leaders to the respective groups of all non-leader women or all non-leader men (or vice versa, for that matter) because only a small subset of each of these groups is likely to become leaders. This limitation can be mitigated by comparing findings on leaders with existing findings on similar group differences from non-leader samples. Thus, to the extent that the findings on leaders are consistent with the findings of other sex difference studies (which they generally tended to be), the sampling problem and range restriction of focusing only on leaders is mitigated.

The fact that we stressed sex differences does not mean that there is no individual variation within the sexes, overlaps between the sexes, or individual plasticity (cf. Bateson and Gluckman, 2011; Del Giudice, 2019; Garfield et al., 2020). It also does not justify or prescribe unequal treatment between the sexes. It is possible that when men and women work together, they can form stronger teams by combining their specific skills, perspectives, and psychological strengths (e.g., Kruger, 2008; Hessami and da Fonseca, 2020). Both men and women are able to learn from each other’s respective leadership styles, thereby broadening their leadership repertoires (Appelbaum et al., 2003; Garfield et al., 2019b). What is more, despite the relative phylogenetic inertia in mammalian leadership patterns, it is also possible that humans can “rise above” their biological history and create social conditions which favor meritocratic leadership regardless of sex (cf. Smith et al., 2020), although gender-based quotas *per se* are likely to have several counterproductive consequences in some contexts. These can include such quota-driven outcomes as creating tension, fostering resentment, impeding collaborative activities, increasing processes of social categorization, intergroup biasing, and competition, being perceived as unfair, bereaving those elected by quotas of their legitimacy and the recognition of their own achievements (Madison, 2019; Euchner and Frech, 2020), and sometimes even adversely affecting collective performance (Yang et al., 2019; though see Liu et al., 2014).

## Conclusion

Evolutionary science—coupled with a recognition of the proximate neurodevelopmental mechanisms and psychobehavioral predispositions reviewed above—has considerable integrative power in explaining sex differences in and out of politics during a pandemic (Figure 1). The research synthesis provided in this article can foster new biopsychosocial research on the ways in which men and women differ in crisis leadership, which psychobehavioral traits those leadership differences are based on, and how the differences can be facultatively harnessed in different ecological and sociopolitical contexts to potentially benefit whole societies. Current evidence indicates that against the invisible viral foe that can bring nations to their knees, the strategies of feminine care-takers and health “worriers” rather than those of masculine risk-taking “warriors” may bring more effective and humanitarian outcomes. We hope that the evolutionary–developmental approach presented in this article contributes to the scientific understanding of sex differences in leadership, inspiring broader consilience across evolutionary science, psychology, political science, anthropology, and developmental, cognitive, and behavioral neuroscience.

## Author Contributions

SL drafted the manuscript and prepared the data visualization and all Figures. MV reviewed the manuscript critically for intellectual content and conceptualized the sexually dimorphic leadership specialization hypothesis. SL and MV wrote, revised, and approved the final manuscript. Both authors contributed to the article and approved the submitted version.

## Conflict of Interest

The authors declare that the research was conducted in the absence of any commercial or financial relationships that could be construed as a potential conflict of interest.

## Footnotes

1 We note the publication of a third cross-national study by Windsor et al. (2020), which did not find significant differences in the proportion of deaths for the group of countries with female leaders relative to the group of countries with male leaders. This null finding could have been partially driven by the low statistical power, as Windsor et al. (2020) included only 12 female-led countries (and 155 male-led countries) in their analyses.

2 Data on total COVID-19 deaths and COVID-19 tests per 1 million inhabitants up to October 30th 2020 were collected from the Worldometer site (https://www.worldometers.info/coronavirus/#countries). Human Development Index data were collected from United Nations Development Programme (http://hdr.undp.org/en/content/human-development-index-hdi). The Human Development Index is a composite index of life expectancy at birth, education, and per capita income.

3 That Belgium is such a stark outlier amongst women-led countries could be because Belgium’s high numbers may have been driven by including also suspected (rather than only confirmed) cases in the total count of COVID-19 deaths (Windsor et al., 2020).

4 Though otherwise Noem’s approach may have been somewhat lax owing to her refusal to mandate mask use.

5 The male leaders were: Jair Bolsonaro of Brazil, Boris Johnson of the United Kingdom, Narendra Modi of India, Andrés Manuel López Obrador of Mexico, Alexander Lukashenko of Belarus, Donald Trump of the United States, Recep Tayyip Erdogan of Turkey, Vladimir Putin of Russia, and Gurbanguly Berdimuhamedow of Turkmenistan.

6 These effect sizes are respectively from Schmitt et al. (2008) and Allen and Robson (2020, based on the reported raw means and standard deviations). Notably, others have argued that sex differences in conscientiousness are confined to just some of its components (Del Giudice et al., 2012).

7 Many of the papers on the evolution of leadership (e.g., von Rueden et al., 2018; Garfield et al., 2019b) or on the evolution of sex differences (e.g., Archer, 2019) do not provide an explicit definition of ‘status’. Some papers do define ‘status’, but the definitions are not always similar. For example, in Hays (2013), ‘status’ was defined as the extent to which one is respected by others. In von Rueden et al. (2011), ‘social status’ was defined as relative access to contested resources within a social group. Garfield et al. (2019a) defined status/prestige as an individual’s value based on subjective evaluations by the group. For this reason, we have not relied on a single definition of ‘status’, but have chosen instead to highlight variation and/or ambiguity in its usage in relevant literature.

8 In the long run, it is possible that the best population-health response is also the best economic response, as a society that has eliminated the virus may be better able to resume healthy economic activity. This remains to be empirically confirmed.

9 Whether similar or opposite patterns are seen in cross-cultural variation in women’s motivation to engage in leadership remains to be determined.

10 Traits other than resources and status may be more beneficial for women when acquiring extra-pair matings.

11 A limitation of these findings is that the women who were coded as leaders in that study did not necessarily have a formal leadership position, but may have gained their influence because of their marriage to a high-status male (Garfield and Hagen, 2020), making it difficult to ascertain the direction of causality between their influence and marital status.
